# Melanoma-restricted genes

**DOI:** 10.1186/1479-5876-2-34

**Published:** 2004-10-15

**Authors:** Ena Wang, Monica C Panelli, Katia Zavaglia, Susanna Mandruzzato, Nan Hu, Phil R Taylor, Barbara Seliger, Paola Zanovello, Ralph S Freedman, Francesco M Marincola

**Affiliations:** 1 Immunogenetics Section, Department of Transfusion Medicine, Clinical Center, National Institutes of Health, Bethesda, Maryland 20892, USA; 2 Department of Oncology and Surgical Sciences, Oncology Section, University of Padova, Padova, Italy; 3 Cancer Prevention Studies Branch, Center for Cancer Research, National Cancer Institute, National Institutes of Health, Bethesda, MD 20892, USA; 4 Institute of Medical Immunology, Martin Luther University Halle-Wittenberg, 06112 Halle, Germany; 5 Department of Gynecologic Oncology, The University of Texas, MD Anderson Cancer Center, Houston, TX, USA

## Abstract

Human metastatic cutaneous melanoma has gained a well deserved reputation for its immune responsiveness. The reason(s) remain(s) unknown. We attempted previously to characterize several variables that may affect the relationship between tumor and host immune cells but, taken one at the time, none yielded a convincing explanation. With explorative purposes, high-throughput technology was applied here to portray transcriptional characteristics unique to metastatic cutaneous melanoma that may or may not be relevant to its immunogenic potential. Several functional signatures could be identified descriptive of immune or other biological functions. In addition, the transcriptional profile of metastatic melanoma was compared with that of primary renal cell cancers (RCC) identifying several genes co-coordinately expressed by the two tumor types. Since RCC is another immune responsive tumor, commonalities between RCC and melanoma may help untangle the enigma of their potential immune responsiveness. This purely descriptive study provides, therefore, a map for the investigation of metastatic melanoma in future clinical trials and at the same time may invite consideration of novel therapeutic targets.

## Background

Human metastatic cutaneous melanoma relative to other common solid tumors shares with renal cell cancer (RCC) the well deserved reputation of being responsive to immune manipulation [1,2]. However, the reason(s) for this phenomenon remain(s) largely unknown [3]. Possibly, metastatic cutaneous melanoma is endowed compared to other tumors with a wealth of "tumor rejection" antigens of unique immunogenic potential. Indeed, the ease in which tumor infiltrating lymphocytes recognizing autologous tumor cells can be isolated from melanoma metastases suggests an extraordinary ability of melanoma cells to elicit cognitive T cell responses [4]. In addition, the broad repertoire of melanoma-associated antigens so far discovered largely outnumbers that of other tumors suggesting a stronger immunogenicity of this cancer [5-7]. This explanation, however, contrasts with the paucity of RCC-specific antigens described and the relative difficulty of expanding tumor infiltrating lymphocytes from RCC that can recognize autologous cancer cells. Yet, RCC is somehow also responsive to immune therapy [2,8]. suggesting that explanations other than solely the identity of T cell epitopes should be considered.

We have previously shown that the microenvironment of a subgroup of melanoma metastases expresses at the transcriptional level an array of biologically active factors that may influence both the innate and the adaptive arm of the immune system [9]. We have also observed that subcutaneous melanoma metastases likely to respond to immunotherapy have a different genetic profile than those unlikely to respond to therapy [10]. This genetic profile differs particularly in expression of immunologically relevant genes suggesting that melanoma metastases that respond to therapy are conditioned to respond even before therapy by an immunologically active environment. These pilot studies encouraged us to collect a large series of melanoma metastases and analyze their genetic profile to search for molecular signatures specific for this tumor entity compared with other less immunogenic cancers. The lack of clinical information limited this study to a descriptive analysis of the molecular signatures characteristic of melanoma that could serve as a map for future studies on this subject. In addition, the application of high-throughput technology to identify transcriptional characteristics unique to metastatic cutaneous melanoma may define novel targets which can be employed for further analysis. . Several signatures were identified descriptive of immune or other biological functions that might be relevant to immune responsiveness. Furthermore, a comparison of the transcriptional profile of metastatic melanoma with that of a library of available primary RCC identified several genes co-coordinately expressed by the two tumor types. Since RCC represents another immune responsive human tumor it is possible that commonalities with melanoma may reveal, in the future, the secret of immune responsiveness. This purely descriptive study provides, therefore, a map of markers for the investigation of metastatic melanoma in novel clinical trials and may invite consideration of novel therapeutic targets.

## Results and Discussion

### Differences between the transcriptional profile of melanoma metastases and other solid tumors

**Figure 1:**
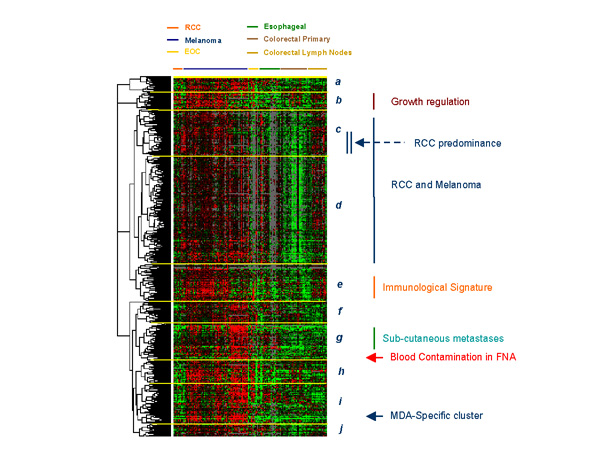
Eisen's clustering based on 2,044 genes up-regulated in metastatic melanoma lesions compared with all other tumors. Signatures include growth regulation (maroon vertical bar); a signature of genes similarly expressed by melanoma and RCC (blue vertical bar) including a sub-cluster of genes predominantly expressed by RCC (double vertical blue bar and blue arrow); an immunological signature (orange vertical bar); a signature specific for genes predominantly expressed by cutaneous and subcutaneous melanoma metastases (green vertical bar); gene related to blood contamination in fine needle aspirates (FNA; red arrow) and a signature specific for melanoma differentiation antigens (MDA; blue arrow). Genes were identified by a two-tailed Student's *t *test comparing all melanoma lesions with other tumors (with the exception of RCC) applying as cut off of significance a p_2_-value < 0.001. Up-regulation was defined as a positive value after subtracting the average of other tumor samples Cy5/Cy3 ratios from that of melanoma samples.

We first identified genes differentially expressed between 69 melanoma samples and 87 samples obtained from available primary or metastatic solid tumors (Table I). RCC samples were excluded from the statistical comparison because this tumors share immune responsiveness with metastatic melanoma and, therefore, were considered separately from non-immunogenic tumors. Differential expression was defined significant at a p_2_-value ≤ 0.001 (unpaired two-tailed Student *t *test). This test identified 4,658 cDNA clones differentially expressed between melanoma metastases and tumors of other histology (see Additional file 1). Non parametric Wilcoxon test yielded comparable results in terms of number and identity of differentially expressed genes (data not shown). Permutation analysis strongly supported the significance of these findings. Approximately half of the differentially expressed clones (2,044) were up-regulated in melanoma metastases relative to other tumors and the remaining 2,614 clones were down-regulated. Up-regulation was defined as a positive value after subtracting the average ratio of other tumors from that of melanoma lesions (Figure 1). Down-regulation was considered a negative value resulting from the same formula. A large proportion of the genes down-regulated in melanoma relative to other tumor were lineage specific and reflected its unique ontogeny from the neuroectoderm while the tumors studied were mostly of epithelial origin. We have previously described the weight that ontogeny may play in balancing the transcriptional profile of RCC [11]. Unfortunately, for this type of analysis to be conclusive availability of matched normal tissues is required which is not as readily achievable in the case of melanoma due to the scattered distribution of normal epithelial melanocytes within the skin layers. The complete list of the 4,658 genes differentially expressed by melanomas is available at 

**Table 1:** Samples used for the analysis presented in the same ordered displayed in the supervised analyses.

Histology	Location	# of Specimens	Source
RCC	Primary	14	Mainz University, Germany
Melanoma	Primary	1	Padua University, Italy
Melanoma	In Transit Metastases	3	Padua University, Italy
Melanoma	Cutaneous Metastases	7	Padua University, Italy
Melanoma	Lymph Node Metastasis	35	Padua University, Italy
Melanoma	Visceral Metastases	2	Padua University, Italy
Melanoma	Cutaneous Metastases (FNA)	21	NCI, NIH, Bethesda, USA
EOC	Primary	15	MD Anderson CC, Houston, TX, USA
Soft Tissue Sarcoma	Primary	3	Tissue Network, Philadelphia, PA, USA
Endometrial Cancer	Primary	1	Tissue Network, Philadelphia, PA, USA
Laryngeal Cancer	Primary	1	Tissue Network, Philadelphia, PA, USA
Breast Cancer	Primary	2	Tissue Network, Philadelphia, PA, USA
Colon Adeno-Carcinoma	Primary	1	Tissue Network, Philadelphia, PA, USA
Esophageal Carcinoma	Primary	12	NCI, NIH, Bethesda, USA,
Colorectal Carcinoma	Primary	35	University of Pisa, Italy
Colorectal Carcinoma	Lymph Node Metastasis	16	University of Pisa, Italy
Colorectal Carcinoma	Hepatic Metastasis	1	University of Pisa, Italy
Total Specimens		180	

RCC = Renal Cell Carcinoma; FNA = Fine Needle Aspirates; EOC = Epithelial Ovarian Cancer;

### Signature-specific genes

**Figure 2:**
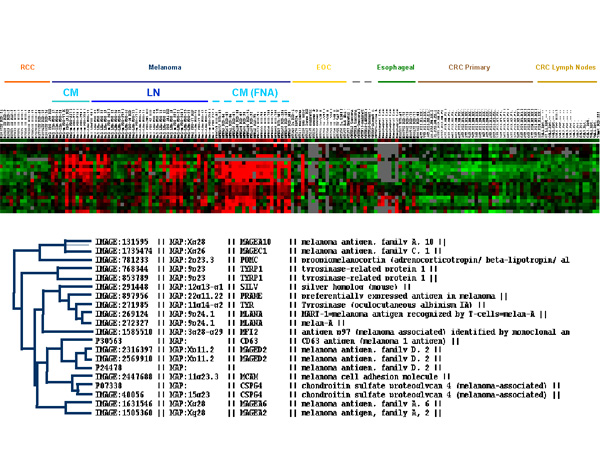
Eisen's clustering of genes already reported to be preferentially expressed by melanomas. The analysis was performed on 180 cancer samples as described in the Results section and ordered according to Table 1. In particular, renal cell cancer (RCC, orange), melanoma (blue), Epithelial Ovarian Cancer (EOC, yellow), Esophageal Cancer (green), Primary Colorectal Cancer (CRC, dark brown) and lymph nodal metastases of CRC (light brown) are shown. Melanoma samples are further subdivided in cutaneous metastases (CM, light blue) from frozen sections (continuous line) or fine needle aspirates (FNA, dashed line) and lymph nodal metastases (LN, darker blue line). Below is the distance among the various genes based on Eisen's clustering.

Several signatures representing genes preferentially expressed by melanomas were identified that could be partially linked to specific gene functions. Those signatures were segregated according to unsupervised gene rearrangement based on the Eisen's clustering method. The first cluster (**cluster *a***, Figure 1) included 76 clones of which 63 were named corresponding to 53 genes. A subset of genes in this cluster were commonly up-regulated in melanoma and RCC including *enolase 2 *(*neuronal γ-enolase*) which is a previously described serum marker of RCC also associated with renal carcinogenesis [11-13]. Differential expression of enolase-2 between melanoma and other cancers with the exception of RCC reached a significance of 5 × 10^-7 ^and 1 × 10^-6 ^for two clones representing this gene (Student's *t*-test p_2 _value). Overall, this cluster was enriched of genes associated with active cellular metabolism and included only few genes of previous known relevance to melanoma with the exception of a member of the melanoma antigen family D (*MAGED2*, Table 2 and Figure 2). **Cluster *b ***included 91 clones (72 named representing 65 distinct genes) predominantly associated with growth regulation and apoptosis. Among the genes included in this cluster was *BNIP3L *(BCL2/Adenovirus E1B interacting protein like-3, *t*-test p_2_-value = 2 × 10^-7 ^for both cDNA clones representing this gene) that we have previously reported to be associated with the immune responsiveness of melanoma metastases [10]. Two large and related clusters (**cluster *c *and *d***) included 262 and 613 clones, respectively (112 and 296 named corresponding respectively to 110 and 289 genes). These clusters were characterized by a high density of unnamed clones and by relatively low Cy5/Cy3 ratios. However, it should be noted that these clusters may be of particular interest because the gene expression profile was similar between melanoma and RCC tumors suggesting that some of these genes may conceal the enigma of immune responsiveness. In particular, a relatively sizable sub-cluster was noted with genes predominantly up-regulated in RCC but also expressed by melanoma lesions compared with other tumors (**Blue arrow and double vertical bar**, Figure 1). Genes concomitantly up-regulated in melanoma and RCC will be separately discussed later, however, it is important to note that this cluster included *JAK-1 *(*t*-test p_2_-value = 2 × 10^-5^) that was previously also reported in association with melanoma immune responsiveness to interleukin-2-based immunotherapy [10]. *JAK-1 *was recently linked to the apoptotic role that interleukin-24 (melanoma differentiation associated gene-7: *MDA 7*) may exert on melanoma cells [14]. The following cluster (**cluster *e***) included 208 clones (151 named representing 143 different genes) predominantly associated with immune function. This immune signature was underlined by the high prevalence of expression of these genes in samples obtained from lymph node metastases whether from melanoma or colorectal primaries. **Cluster *f ***integrated 129 clones (91 named representing 87 distinct genes) including a mixture of genes with disparate functions difficult to categorize into a predominant pattern. This group also included *APPBP1 *(amyloid β precursor protein; *t*-test p_2_-value = 1 × 10^-4^) which was previously reported in association with melanoma immune responsiveness [10]. *APPBP1 *is a recently discovered epidermal growth factor that regulates dendrite motility and melanin release in epidermal melanocytes and melanoma cells [15]. It is possible that some of its functions may have an indirect effect in modulating the immunological profile of subcutaneous metastases. **Cluster *g ***included genes preferentially up-regulated in subcutaneous melanoma metastases known to be more responsive to immunotherapy with interleukin-2 [16]. This cluster included 201 clones (142 named representing 132 genes). Among the genes representative of this cluster were two classic melanoma associated genes (*PRAME *and tyrosine-related protein-1; *TRP-1*). In a small proportion, this cluster included a group of genes only over-expressed in fine needle aspirates (FNA) and generally expressed by circulating cells revealing blood contamination of FNA material (**red arrow**, Figure 1). **Cluster *h ***included 131 clones (102 named representing 99 genes). **Cluster *I ***included 222 clones (171 named identifying 155 genes) with most of the melanoma differentiation antigens (MDA) clustering in close proximity with the exception of the *TRP-1 *already discussed in **cluster *g***. Interestingly, this cluster was also highly enriched of genes associated with ribosomal function and active translation. Furthermore, it included the melanocyte master regulator *MITF *(*t*-test p_2_-value for two respective cDNA clones = 2 × 10^-15 ^and 8 × 10^-14^) which has been shown to modulate lineage survival and melanoma cell viability through interaction with the anti-apoptotic protein BCL2 [17]. *MITF *was coordinately expressed with several genes associated with calcium and other solute metabolism including cytochrome p450 (*t*-test p_2_-value for two respective cDNA clones = 3 × 10^-12 ^and 7 × 10^-13^) solute carrier family 7 (*t*-test p_2_-value for two respective cDNA clones = 2 × 10^-10 ^and 2 × 10^-6^), G protein coupled receptor 56 (*t*-test p_2_-value = 2 × 10^-7^) and calpain 3 (*t*-test p_2_-value = 6 × 10^-15^), a calcium-regulated gene found to be highly expressed in melanoma cells [18]. Finally, **cluster *J ***included 88 clones of which the 62 named identified 58 genes.

**Table 2:** Genes of known association with melanoma

**Clone ID**	**Chromosomal Location**	**Name**	**AVERAGE**			***t*-test (p_2_-value)**	
			**RCC**	**MEL**	**Other**	**RCC vs MEL**	**MEL vs Other**
			
**Cluster *a***							
2569910	Xp11.2	**MAGED2**	-0.22	0.4	-0.28	7.10E-03	8.00E-07
316397	Xp11.2	**MAGED2**	-0.24	0.41	-0.29	5.60E-04	3.00E-07
P24478	Xp11.2	**MAGED2**	-0.21	0.31	-0.22	5.10E-03	1.00E-06
**Cluster *d***							
1735474	Xq26	**MAGEC1**	0.03	0.25	-0.26	3.40E-02	6.80E-06
131595	Xq28	**MAGEA10**	-0.03	0.28	-0.24	6.20E-02	1.80E-04
1505360	Xq28	**MAGEA2**	-0.88	0.94	-0.76	6.00E-12	2.00E-10
**Cluster *e***							
781233	2p23.3	**POMC**	-0.03	0.17	-0.15	2.20E-01	1.40E-05
**Cluster *g***							
897956	22q11.22	**PRAME**	-0.62	1.33	-1.05	1.60E-06	1.00E-18
853789	9p23	**TYRP1**	-0.82	0.59	-0.3	7.70E-06	8.00E-04
768344	9p23	**TYRP1**	-0.7	0.8	-0.61	1.60E-07	1.80E-06
40056	15q23	**CSPG4**	-0.32	0.69	-0.53	2.20E-04	2.70E-09
P07338	n.a.	**CSPG4**	-0.63	0.78	-0.61	8.50E-05	5.70E-09
2447688	11q23.3	**MCAM**	0.21	0.69	-0.6	8.70E-02	3.40E-09
1585510	3q28-q29	**MFI2 (p97)**	-0.48	0.59	-0.44	1.10E-03	5.70E-07
**Cluster *i***							
P30563	n.a.	**CD63**	-0.66	0.7	-0.44	8.20E-08	1.90E-09
1631546	Xq28	**MAGEA6**	-0.55	0.35	-0.22	1.30E-08	5.60E-04
291448	12q13-q1	**SILV (gp100)**	-1.31	1.55	-1.16	4.90E-16	5.00E-15
271985	11q14-q2 || TYR	**Tyrosinase**	-1.37	1.73	-1.38	6.30E-18	2.90E-18
272327	9p24.1	**Melan-A**	-0.76	1.19	-0.95	2.40E-08	1.80E-16
269124	9p24.1	**Melan-A**	-0.65	1.21	-0.99	5.30E-09	2.70E-16

### Genes previously recognized to be associated with melanoma

Genes previously described to be preferentially expressed by melanoma lesions were confirmed to be so at a very high level of significance (Figure 2). Exceptions included AIM-1, CXCL-1 (GRO-α), D2S448 and MAGEA1 which are all significantly more expressed by tumors other than melanomas. Interestingly, different types of melanoma associated genes displayed a different pattern of expression with MDA (tyrosinase, gp100/PMel17 and MART-1/MelanA) being co-coordinately expressed in close proximity to each other in **cluster *I ***and *MAGE *family genes preferentially expressed in **cluster *d ***(Table II). **Cluster *g ***included a number of genes whose expression had been previously associated with melanoma including preferentially expressed antigen in melanoma (*PRAME*) and the tyrosine-related protein-1 (*TRP-1*). When the melanoma associated genes were studied alone, *PRAME *clustered close to the other MDA believe to be involved in the pigmentation process (tyrosinase, MART-1/Melan and gp100/PMel17). This is of particular interest because PRAME has been also reported to be highly expressed in other cancers of ectodermic origin such as medulloblastoma and neuroblastoma suggesting a link between ectoderm and pigmentation [19,20]. The coordinated expression of MDA suggests that their down-regulation or loss of expression during melanoma progression may be related to a central regulatory pathway not as yet identified. Indeed in previous studies [21-25], we noted that loss of expression of MART-1/Melan A paralleled that of gp100/PMel17 (*SILV*) in melanoma metastases while genes of the MAGE family manifested an independent behavior [25]. This finding may have important repercussions in the design of antigen-specific immunization protocols and at the same time may complicate the interpretation of tumor antigen loss variant analysis by broadening loss of expression to antigens other than those targeted by a given therapy.

### Immunological Signature

**Figure 3:**
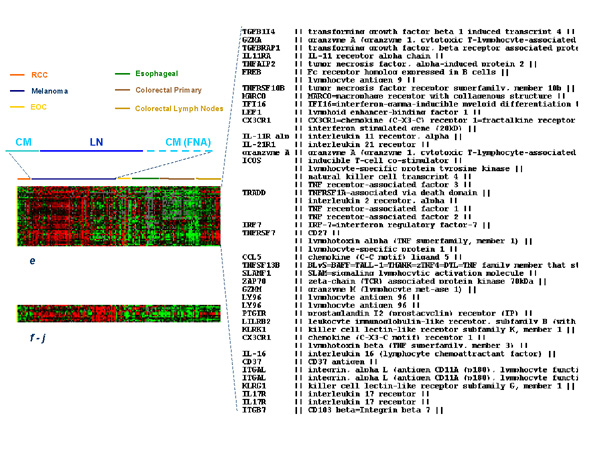
Eisen's clustering of immunologically relevant genes selected from **clusters *e ***and ***f-j***. To the right the identity of the genes included in **cluster *e ***is shown.

The large majority of genes associated with immune function were included in **cluster *e***. These genes appeared up-regulated in lymph node metastases of melanoma as well as those from colorectal primaries suggesting that their expression results from lymphoid cell infiltration (Figure 3). The same genes were up-regulated in a significant proportion of subcutaneous melanoma metastases suggesting that a strong and active infiltrate of immune cells is present in these tissues. In fact, most of the genes included in **cluster *e ***were significantly up-regulated in 10 cutaneous/subcutaneous melanoma lesions compared to 70 primary cancers of other histology (Table III shows a selection of the most significantly up-regulated genes in cutaneous/subcutaneous melanomas). Of interest is the observation that several of the genes up-regulated in these lesions are clustered in specific chromosomal locations with a high predominance of genes located in position 6p21.3, 11p11.2, 19p13 and 19q13. Among the immunologically-related genes specifically up-regulated in subcutaneous melanoma metastases, some are of particular interest because of their known relationship with effector T cell function. In particular, we find interesting that *NK4*, an anti-angiogenic factor released by natural killer cells [26], was constitutively expressed by cutaneous melanomas. We found this gene to be associated with regression of a melanoma metastasis during interleukin-2 therapy [27] and to be one of the genes most frequently up-regulated during activation of antigen-specific T cells *in vitro *[28,29]. Of interest was also the constitutive expression of *CD27 *a co-stimulatory member of the TNF receptor family strongly associated with cell activation [30,31]. The expression of *CX3CR1 *a gene constitutively expressed by natural killer cells that makes them sensitive to chemo-attraction by CXCL12 and CXC3L1 [32] may be an explanation for a preferential localization of these effector cells in melanoma lesions. In particular, this finding suggests that the microenvironment of melanoma metastases is rich of fractalkine (CX3CL1) which is a potent chemo-attractant released by endothelial cells stimulated by interferons [33]. Overall, the presence of these and other (*KLRG1*; killer cell lectin like receptor subfamily G, member 1 and *KLRK1*; killer cell lectin like receptor subfamily K, member 1 and the interleukin-21 receptor) natural killer cell-related genes suggests potent chemo-attraction toward natural killer cells by the tumor micro-environment of subcutaneous and cutaneous melanoma metastases. This is also emphasized by the high expression of interleukin-21 receptor which is usually expressed by natural killer cells and stimulates their cytolytic activity upon ligation with interleukin-21 produced by activated T cells [34,35]. The constitutive expression of interferon regulatory factor (*IRF*)-7 implicated in the amplification of the innate immune response [36] through interactions with the NF-κB pathway [37] may lead to the activation of various types of type I interferons [38]. More puzzling is the constitutive expression of interleukin-16, a pleiotropic cytokine with predominant chemo-attractant activity for CD4+ T cells [39] and CD4+ eosinophils [40]; a relationship between this cytokine and melanoma metastases has never been observed before. In summary, the immunological signature portrayed by subcutaneous melanoma metastases is that of an active innate immune response centered on natural killer cells. More broadly, the preferential expression of genes with immune function in melanoma lesions compared with other tumors suggests that this cancer is constitutively immunologically active and this status may predispose metastatic melanoma to respond to general or antigen-specific immune manipulation.

**Table 3:** Immune-relevant genes specifically up-regulated by sub-cutaneous melanomas

**Clone ID**	**Location**	**Gene**	**AVERAGE**		***t*-test (p_2_-value)**	
			**SQ**	**Other**	**SQ vs Oth**	**Me vs Oth**
295868	1p34	LAPTM5	0.43	-0.68	2.00E-04	3.00E-04
P37265	1p34.3	LCK	0.55	-0.58	1.00E-04	6.00E-06
2563224	1p36.2	PIK3CD	0.8	-0.88	3.00E-07	5.00E-15
842871	1q12	PDE4DIP	0.58	-0.21	5.00E-04	9.00E-05
773509	1q21.3	SNX27	1.05	-0.88	9.00E-11	7.00E-16
701332	1q22	IFI16	0.25	-0.39	2.00E-04	1.00E-05
472009	1q42.1	DISC1	0.35	-0.26	1.00E-07	9.00E-08
746229	2q11.2-q	MAP4K4	0.15	-0.24	7.00E-04	6.00E-05
840466	2q12-q13	MARCO	0.34	-0.28	3.00E-05	2.00E-04
328542	2q24-q3	GALNT3	0.51	-0.4	4.00E-04	1.00E-03
825715	2q37.1	SP110	0.71	-0.59	9.00E-07	3.00E-09
283023	3p21	CX3CR1	0.31	-0.34	2.00E-05	4.00E-10
1605539	4p16.3	IDUA	0.27	-0.29	4.00E-06	6.00E-06
724932	5q35	GRK6	0.43	-0.19	4.00E-05	3.00E-05
753587	6p21.3	BTN3A3	0.49	-0.43	2.00E-05	5.00E-06
753236	6p21.3	TAP2	0.31	-0.42	2.00E-04	1.00E-07
752557	6p21.3	GPSM3	0.42	-0.42	1.00E-04	3.00E-06
2549448	6q21	FYN	0.6	-0.45	1.00E-07	2.00E-07
2306953	8q13.3	LY96	1.01	-0.32	3.00E-06	2.00E-08
645332	10p12	NEBL	0.23	-0.23	6.00E-04	2.00E-04
1631391	11p11.2	BHC80	0.35	-0.29	8.00E-04	4.00E-04
686164	11p11.2	DGKZ	0.44	-0.21	2.00E-04	4.00E-04
487115	11p11.2	PTPRJ	0.78	-0.4	3.00E-09	5.00E-07
151430	11p13	CD44	0.71	-0.22	1.00E-03	2.00E-05
740117	11p15.5	IRF-7	0.53	-0.3	4.00E-05	3.00E-04
P33303	11p15.5	LSP1	0.55	-0.4	1.00E-03	2.00E-05
1850690	11q23.3	BLR1	0.36	-0.36	6.00E-05	2.00E-04
2120815	12p12-p1	KLRG1	0.51	-0.44	1.00E-04	5.00E-04
34637	12p13	CD27	0.72	-0.54	4.00E-04	1.00E-04
1517162	12p13.2-	KLRK1	0.55	-0.53	5.00E-04	6.00E-04
1569551	12q13.11	CSAD	0.37	-0.45	2.00E-04	6.00E-06
429186	13q21.33	LMO7	0.41	-0.37	5.00E-06	4.00E-19
P41256	15q26.3	IL-16	0.64	-0.58	4.00E-05	2.00E-06
P14913	16p11	IL-21R1	0.48	-0.43	6.00E-06	6.00E-04
P07382	16p11.2	ITGAL	0.55	-0.48	2.00E-05	3.00E-06
P12753	16p13.3	NK4;	0.32	-0.3	4.00E-05	3.00E-06
206795	17p	ASGR2	0.57	-0.53	8.00E-05	6.00E-06
488575	17p11.2	ULK2	0.35	-0.17	3.00E-06	1.00E-04
155717	17q23	CD79B	0.44	-0.41	3.00E-05	2.00E-11
156343	17q24.2	MAP3K3	0.62	-0.46	4.00E-06	7.00E-11
P38436	17q25	CARD14	0.54	-0.33	4.00E-08	2.00E-08
1551273	19p12	MEF2B	0.19	-0.21	7.00E-05	1.00E-09
814377	19p13.1	BRD4	0.9	-0.7	6.00E-06	2.00E-16
2010562	19p13.3	MYO1F	0.55	-0.62	9.00E-04	7.00E-05
824384	19p13-q1	CD37	0.64	-0.74	1.00E-03	7.00E-04
788272	19q13.1	CLC	0.61	-0.64	7.00E-06	3.00E-06
815239	19q13.13	ARHGEF1	0.38	-0.42	3.00E-05	1.00E-04
683276	19q13.33	CARD8	0.86	-0.64	1.00E-08	3.00E-05
277906	19q13.4	LILRB1	0.89	-0.51	4.00E-04	5.00E-04
202897	19q13.4	LILRB2	0.72	-0.61	4.00E-04	1.00E-06
2072768	20q12	NCOA3	0.69	-0.36	4.00E-06	2.00E-05

SQ = Average Cy5/Cy3 ratios of10 frozen samples from cutaneous and subcutaneous metastases as described in Table I. Oth = Sample from 80 primary tumors other than melanoma and RCC with the exclusion, in this table of lymph nodal metastases from colorectal cancer (see Table I). Complete and extended gene name is available at

A second and smaller group of immunologically-related genes was identified and included genes that segregated separately in **clusters *f ***to ***j***. These genes had an expression profile opposite to the immune-related genes seen included in **cluster *e ***and appeared over-expressed in subcutaneous compared to lymph node metastases. Two granzyme-related genes were found strongly up-regulated in **cluster *e ***including granzyme A and M. This observation contrasted with the increased expression of cathepsin F and L in **cluster *f *to *j ***suggesting an opposite regulation of these genes involved in cell death or survival.

### Subcutaneous metastases-associated genes

It has been reported that subcutaneous metastases of melanoma are more responsive to immunotherapy with interleukin-2 than lymph nodal and visceral metastases [16]. Therefore, we identified genes differentially expressed in the former compared with the latter. Since no material from visceral metastases was available, we limited the comparison to subcutaneous versus lymph nodal metastases. Overall, **clusters *g* - *j ***appeared to demonstrate a preferential expression of genes in subcutaneous metastases independent of the technique used for biopsy (excision versus FNA). In particular, **cluster *g ***contained a small node of 47 clones highly expressed in subcutaneous metastases that included *PRAME *and *TRP-1*. This cluster also included the renal tumor antigen *RAGE *which has been previously shown to be highly expressed by melanomas [41] and melanophilin and the s100 protein often associated with clinical parameters in melanoma [42]. Interestingly, closely linked to *PRAME *was the pattern of expression of the serine/threonine-specific protein kinase *B-RAF*. This gene is mutated in approximately 70 % of melanomas and it is often over-expressed [43]. Although several of these genes had been associated with melanoma their co-ordinate expression has never been previously appreciated. Overall, the identity of the genes over-expressed in subcutaneous metastases did not offer an obvious explanation for the increased immune responsiveness of these lesions and more extensive understanding of their relationship will be necessary in the future.

### Genes differential expressed by melanoma and RCC samples compared with other solid tumors

**Figure 4:**
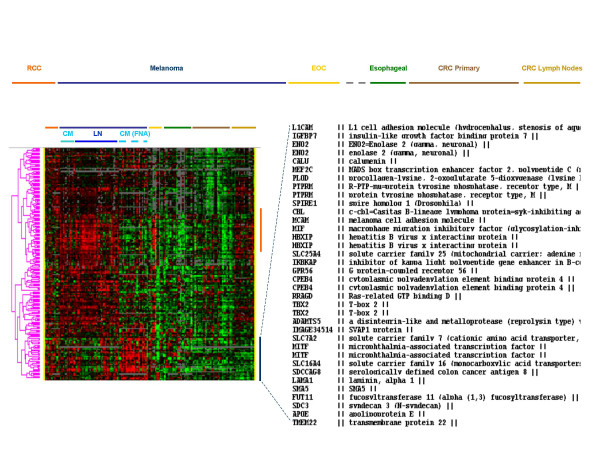
Eisen's clustering of genes similarly expressed by RCC and melanoma lesion. To the right the identity of genes most prominently expressed by RCC lesions and cutaneous or subcutaneous melanoma lesions is shown.

We then pooled together melanoma and RCC samples data to identify genes commonly expressed by these tumors and not by tumors of other histology. Significance was assessed by a two-tailed unpaired student's *t *test and identified 4,221 genes at a cut off p_2_-value of ≤ 0.001. The data set was then filtered using the Cluster Program (Stanford, CA) selecting genes that were expressed in at least 80 % of the experiments and for which a Cy5/Cy3 log_2 _ratio ≥ 2 was present in at least one experiment. Two-thousand eight hundred and forty-three genes resulted from this filter. Because of the predominance of melanoma lesions (69 melanoma lesions compared to 14 RCC) the genes identified strongly represent differences between melanomas and other tumors. Therefore, we identified among them those that were not differentially expressed between melanoma and RCC samples to identify those genes that are truly uniquely expressed by the two immune responsive cancers. Two-thousand three hundred and fifty-eight genes were expressed similarly between the two types of cancer (at a *t *test p_2_-value > 0.05). A significant number of genes commonly expressed by melanoma and RCC and not by other tumors had no known function (681 genes). The remaining 1, 677 genes were further analyzed by separating those up-regulated from those down-regulated in melanoma and RCC compared with other tumors. The genes up-regulated in RCC and melanoma were considered those with a median Log_Ratio _above 0.3 in either RCC or melanomas (**a selection of these genes is shown in **Table IV). This analysis selected 199 genes. A proportion of genes appeared to be specifically related to lymph nodal and immune infiltration as they were particularly up-regulated in melanoma metastases to lymph nodes and in lymph node metastases obtained from patients with CRC (**orange vertical bar**, Figure 4). These genes included annotations related to immunological function. A set of genes was specifically expressed in cutaneous metastases of melanoma and in RCC and not other tumor samples (**dark blue vertical bar**, Figure 4). These genes included microphtalmia transcription factor *MITF *[17,44]. that has been shown previously to exert a central role in the regulation of transcriptional activity of melanoma cells. Similarly, enolase-2 (previously known to be up-regulated in RCC) [11] was found to be over-expressed in common between the two histologies. This is somewhat surprising since immunohistochemical analysis has used lack of staining for enolase-2 as a reliable method to differentiate malignant melanoma (enolase-2 negative) from Merkel cell carcinoma [45]. It is possible, that although identifiable at the transcriptional level, enolase-2 is not processed into a protein in melanomas. On the other hand, enolase-2 has been shown to be expressed in approximately 90 % of canine oral melanomas [46]. In addition, the macrophage migration inhibiting factor (*MIF*) which is a modulator of cell cycle progression and angiogenesis in melanoma [47,48]. was found co-expressed by melanoma and RCC lesions. *MIF *has modulatory properties on natural killer cell mediated lysis of cancer cells contributing, therefore, to an immune privileged microenvironment in uveal melanoma [49]. Two genes coding for adhesion molecules; L1 cell adhesion molecule (*LCAM*) and melanoma cell adhesion molecule (*MCAM*) were also up-regulated in both lesions and may play an important role in mediating migration of immune cells to the tumor deposits [50]. Finally, it is remarkable that serologically defined colon cancer antigen 8 was specifically expressed by melanoma and RCC while was completely absent in colon cancers underlying the need for a better nomenclature of newly identified genes.

**Table 4:** Selected genes constitutively expressed by RCC and melanoma metastases.

UNIQID	NAME	Extended Name	Median _log2_Cy5/Cy3			Average_log2_Cy5/Cy3			*t*-test*
			RCC	MEL	OTH	RCC	MEL	OTH	p_2_-value
274276	IFIT2	interferon-induced protein with tetratricopeptide repeats 2	0.74	0.30	-0.28	0.54	0.18	-0.23	0.06
191173	ITGB7	integrin, beta 7	0.17	0.33	-0.26	0.14	0.30	-0.26	0.51
191169	FLT3LG	fms-related tyrosine kinase 3 ligand	0.18	0.32	-0.27	0.31	0.19	-0.20	0.53
187264	CORO1A	coronin-like protein p57=actin binding protein p57	0.38	0.13	-0.19	0.47	0.12	-0.17	0.16
189684	SP110	SP110 nuclear body protein	0.22	0.60	-0.56	0.26	0.44	-0.42	0.27
279561	TNFRSF7	CD27	-0.06	0.65	-0.39	-0.09	0.45	-0.35	0.07
279871	CD37	CD37 antigen	0.09	0.56	-0.56	0.33	0.47	-0.38	0.72
276143	TAP2	transporter 2	0.23	0.33	-0.21	0.27	0.31	-0.24	0.86
281103		sialic acid binding Ig-like lectin 7=D-siglec=expressed in dendritic cells	0.33	0.26	-0.34	0.27	0.24	-0.24	0.86
279699	BTK	btk = Bruton agammaglobulinemia tyrosine kinase ||	-0.04	0.45	-0.28	-0.09	0.34	-0.26	0.05
274604	CST7	cystatin F (leukocystatin) ||	-0.14	0.37	-0.29	-0.07	0.45	-0.34	0.06
281440	ITGB7	CD103 beta=Integrin beta 7 ||	0.36	0.39	-0.25	0.31	0.38	-0.35	0.67
191157	KLRG1	killer cell lectin-like receptor subfamily G, member 1 ||	0.32	0.40	-0.17	0.35	0.23	-0.25	0.39
274016	RASGRP1	RAS guanyl releasing protein 1 (calcium and DAG-regulated) ||	0.48	0.38	-0.29	0.36	0.30	-0.29	0.78
274444	ITGAL	integrin, alpha L (antigen CD11A (p180)|	0.34	0.57	-0.43	0.30	0.33	-0.31	0.88
282504	CX3CR1	chemokine (C-X3-C motif) receptor 1	0.84	0.66	-0.53	0.62	0.51	-0.50	0.76
274267	KLRK1	killer cell lectin-like receptor subfamily K, member 1	0.80	0.33	-0.20	0.71	0.34	-0.38	0.14
187290	LILRB1	LIR-7=PIR homologue|	0.11	0.67	-0.15	0.12	0.49	-0.41	0.20
186380	SLC2A3	solute carrier family 2 (facilitated glucose transporter), member 3	0.14	0.34	-0.21	0.34	0.36	-0.34	0.94
188111	CD3Z	CD3Z antigen, zeta polypeptide (TiT3 complex)	0.09	0.42	-0.30	-0.01	0.35	-0.27	0.10
186528	SLA	SLAP=src-like adapter protein	0.38	0.31	-0.24	0.32	0.33	-0.31	0.98
185279	ASGR2	asialoglycoprotein receptor 2|	0.28	0.61	-0.33	0.17	0.45	-0.40	0.16
187450	LILRB2	leukocyte immunoglobulin-like receptor, subfamily B, member 2	0.10	0.65	-0.45	0.13	0.54	-0.44	0.07
184382	FGR	Gardner-Rasheed feline sarcoma viral (v-fgr) oncogene homolog|	0.57	0.23	-0.29	0.40	0.27	-0.28	0.49
190623	MYO1F	myosin IF|	0.18	0.50	-0.22	0.16	0.46	-0.38	0.24
188800	PILRA	paired immunoglobin-like type 2 receptor alpha	0.16	0.56	-0.08	0.07	0.31	-0.27	0.17
188004	CLC	Charcot-Leyden crystal protein|	0.08	0.51	-0.33	0.08	0.53	-0.44	0.08
186399	PPP3CC	protein phosphatase 3, catalytic subunit, gamma isoform (calcineurin A gamma)|	0.07	0.36	-0.15	0.05	0.21	-0.18	0.17
278997	XLHSRF-1	heat shock regulated 1	-0.12	0.32	-0.09	0.10	0.28	-0.20	0.33
281827	LLT1	lectin-like NK cell receptor	0.46	0.26	-0.33	0.53	0.22	-0.26	0.08
282466	LLT1	lectin-like NK cell receptor	0.22	0.38	-0.23	0.23	0.32	-0.32	0.66
189527	FMNL1	formin-like 1	0.46	0.43	-0.25	0.44	0.24	-0.26	0.32
282550		natural killer cell transcript 4	-0.20	0.41	-0.29	0.00	0.30	-0.24	0.15
282534		B-cell CLL/lymphoma 2	0.05	0.38	-0.38	-0.05	0.33	-0.34	0.05
282477	ICOS	inducible T-cell co-stimulator|	-0.07	0.37	-0.18	-0.07	0.28	-0.26	0.07
282624	granzyme A	granzyme A (granzyme 1, cytotoxic T-lymphocyte-associated serine esterase 3)|	-0.10	0.58	-0.33	-0.16	0.43	-0.31	0.06

* Two-tailed un-paired *t *test between RCC and MEL samples. RCC = Renal cell carcinoma; Mel = melanoma lesions; Oth = all other tumors in the study (see Table I).

This is a descriptive study where genes specifically expressed by melanoma metastases were identified comparing a large collection of samples from patients with metastatic cutaneous melanoma with other primary tumors and lymph nodal metastases. A limitation of the study is the lack of other samples including visceral metastases of melanoma and metastases from tumors of other histology. Nevertheless, we considered useful to compile a list of genes characteristically expressed by subcutaneous and lymph nodal lesions of melanoma for reference purposes and we are willing to provide full information about these genes upon request. In spite of the limitations of this study, few general conclusions could be drawn.

## Materials and Methods

### Tissue procurement

Fourteen primary renal cell carcinoma (RCC) specimens were collected at the Department of Urology of The Johannes Gutenberg-University, Mainz, Germany; one primary melanoma, three in transit metastases, seven cutaneous metastases, thirty-five lymph nodal metastases and two visceral metastases of cutaneous melanoma were collected at the Department of Surgical Sciences, University of Padua, Italy; twenty-one fine needle aspirates of cutaneous melanoma metastases were obtained at the Surgery Branch, National Cancer Institute, National Institutes of Health, Bethesda, MD; seventeen primary epithelial ovarian cancer (EOC) specimens were obtained at the Department of Gynecologic Oncology, MD Anderson Cancer Center, TX; three primary sarcoma, one primary endometrial cancer, one primary laryngeal cancer, two primary breast cancers and one primary colon adeno-carcinoma were obtained from the Tissue Network (Philadelphia, PA); twelve primary carcinomas of the esophageal junction were obtained from the NCI (Division of Cancer Treatment and Diagnosis); thirty-five primary, 16 lymph node metastases and one hepatic metastasis from colorectal adeno-carcinomas were obtained from the Department of Pathology of the University of Pisa, Italy. Specimens were collected as the result of routine operative procedures and portions were frozen for subsequent analysis while the remnant tissue was used for pathological confirmation. Tissue procurement followed standard ethical procedure according to institutional policy. A summary of the specimens studied is presented in Table 1 with their order reflecting their distribution in figures where supervised analyses are shown.

### RNA preparation, amplification and labeling

Total RNA was extracted from frozen material using Trizol reagent according to manufacturer's instructions (Invitrogen, CA) and amplified into anti-sense RNA (aRNA) as previously described [10,27,51,52]. Although the quantity of starting total RNA was in most cases sufficient for cDNA array hybridization, we have shown repeatedly that the fidelity of aRNA hybridization is at least equal and likely superior to total RNA for transcriptional profiling due to lack of contaminant ribosomal and transfer RNA [51,53]. Therefore, we used aRNA to increase consistency of results particularly when low quality total RNA was documented by Agilent Bioanalyzer 2000 (Agilent Technologies, Palo Alto, CA). After amplification the quality of aRNA was tested with the Agilent Bioanalyzer as previously described [52].

Total RNA from peripheral blood mononuclear cells pooled from six normal donor was extracted and amplified to serve as constant reference as previously described [10,27,51,52]. Test and reference RNA were labeled with Cy5 (red) and Cy3 (green) and co-hybridized to a costum-made17.5 K cDNA micro-array . Micro-arrays were printed at the Immunogenetics Section, DTM, CC, NIH with a configuration of 32 × 24 × 23 and contained 17,500 elements. Clones used for printing included a combination of the Research Genetics RG_HsKG_031901 8 k clone set and 9,000 clones selected from the RG_Hs_seq_ver_070700 40 k clone set. The 17,500 spots included 12,072 uniquely named genes, 875 duplicated genes and about 4,000 expression sequence tags.

### Data analysis

All statistical analyses were performed using the log_2_-based ratios normalizing the medial log_2 _ratio value across the array equal to zero. Validation and reproducibility were performed using our internal reference concordance system as previously described [54]. Unsupervised clustering was performed according to the Eisen's Pearson correlation method [55] and visualized with Tree-View software (Stanford University, CA). Genomic portraits were depicted according to the central method for display using a normalization factor as suggested by Ross et al. [56]. Details about different tests are discussed in the respective results section. Identification of tumor-specific genes was performed using un-paired 2-tailed Student's *t *test. The same analyses were performed using un-paired Wilcoxon's non-parametric assessment and provided the same conclusions (not shown). Details of each analysis are presented in the results section.

## Supplementary Material

Additional file 1 AVE Ratio = average Log2 CY5/Cy3 ratio between test and reference sample. The t test p2-value refers to a two-tailed unpaired analysis between the samples mentioned below. RCC = renal cell cancer; MEL = melanoma; Other = tumors other than RCC and melanoma. Click here for file
